# Transcranial focused ultrasound stimulation of motor cortical areas in freely-moving awake rats

**DOI:** 10.1186/s12868-018-0459-3

**Published:** 2018-09-19

**Authors:** Wonhye Lee, Phillip Croce, Ryan W. Margolin, Amanda Cammalleri, Kyungho Yoon, Seung-Schik Yoo

**Affiliations:** Department of Radiology, Brigham and Women’s Hospital, Harvard Medical School, 75 Francis Street, Boston, MA 02115 USA

**Keywords:** Transcranial focused ultrasound, FUS, Brain stimulation, Motor cortex, Wearable headgear, Awake rat, Ketamine/xylazine, Isoflurane

## Abstract

**Background:**

Low-intensity transcranial focused ultrasound (tFUS) has emerged as a new non-invasive modality of brain stimulation with the potential for high spatial selectivity and penetration depth. Anesthesia is typically applied in animal-based tFUS brain stimulation models; however, the type and depth of anesthesia are known to introduce variability in responsiveness to the stimulation. Therefore, the ability to conduct sonication experiments on awake small animals, such as rats, is warranted to avoid confounding effects of anesthesia.

**Results:**

We developed a miniature tFUS headgear, operating at 600 kHz, which can be attached to the skull of Sprague–Dawley rats through an implanted pedestal, allowing the ultrasound to be transcranially delivered to motor cortical areas of unanesthetized freely-moving rats. Video recordings were obtained to monitor physical responses from the rat during acoustic brain stimulation. The stimulation elicited body movements from various areas, such as the tail, limbs, and whiskers. Movement of the head, including chewing behavior, was also observed. When compared to the light ketamine/xylazine and isoflurane anesthetic conditions, the response rate increased while the latency to stimulation decreased in the awake condition. The individual variability in response rates was smaller during the awake condition compared to the anesthetic conditions. Our analysis of latency distribution of responses also suggested possible presence of acoustic startle responses mixed with stimulation-related physical movement. Post-tFUS monitoring of animal behaviors and histological analysis performed on the brain did not reveal any abnormalities after the repeated tFUS sessions.

**Conclusions:**

The wearable miniature tFUS configuration allowed for the stimulation of motor cortical areas in rats and elicited sonication-related movements under both awake and anesthetized conditions. The awake condition yielded diverse physical responses compared to those reported in existing literatures. The ability to conduct an experiment in freely-moving awake animals can be gainfully used to investigate the effects of acoustic neuromodulation free from the confounding effects of anesthesia, thus, may serve as a translational platform to large animals and humans.

**Electronic supplementary material:**

The online version of this article (10.1186/s12868-018-0459-3) contains supplementary material, which is available to authorized users.

## Background

Over the past few decades, various brain stimulation techniques have significantly contributed to enhancing our current understanding of neural/neuronal function and offered non-pharmacological options for the treatment of neurological and neuropsychiatry diseases [1–3]. Approaches, such as deep brain stimulation (DBS) or epidural cortical stimulation (EpCS) [3], allow for stimulating brain regions with excellent spatial specificity, but require invasive surgical procedures. Transcranial direct current stimulation (tDCS) and transcranial magnetic stimulation (TMS) provide non-invasive alternatives to the surgical procedures, but may not reach deep brain areas with a centimeter-scale area for stimulation, limiting spatial specificity [1, 2]. Optogenetic techniques are capable of modulating cellular level activity of the brain [4]; however, the necessary genetic modification of neurons to gain light-sensitivity and limited transcranial penetration of stimulatory light may obstruct its translational application in humans.

Focused ultrasound (FUS) technique allows for the non-invasive, focal delivery of mechanical pressure waves to regional biological tissues [5–7], measuring a few millimeters in diameter and length. The advances in FUS techniques have further enabled the transcranial delivery of acoustic energy to specific regions of the brain [8–10]. This transcranial FUS (tFUS) technique has been utilized for non-invasive functional neurosurgery by thermally ablating localized deep brain structures, whereby the ultrasound waves are delivered at high acoustic intensities [11, 12]. tFUS has also been applied to temporarily open the blood-brain barrier (BBB) when combined with intravascular administration of microbubbles (detailed review can be found in [13]). In addition to these therapeutic potentials, tFUS, given in a train of pulses at a low-intensity (under the threshold for heat generation), has been shown to reversibly modulate regional brain excitability [14–17]. Taking advantage of the exquisite ability to transcranially reach deep brain areas [18, 19] as well as cortical areas [20–25] with high spatial selectivity, low-intensity tFUS has rapidly gained momentum as a new mode of non-invasive brain stimulation [26, 27].

FUS has shown to modulate excitability in motor/visual cortical areas in rabbits [17], stimulated various motor cortices in mice [16, 28–32], suppressed epileptic seizure electroencephalographic (EEG) activities [33], and altered the extracellular neurotransmitter level [34, 35] and anesthesia time in rats [36]. Investigations have also been conducted to study the effect of varying acoustic parameters [37] and spatial profile of neuromodulation using a rat model [38, 39]. Additionally, tFUS has stimulated the motor and visual cortices in sheep and elicited corresponding electrophysiological responses [24]. The majority of these studies, conducted on anesthetized animals, showed a degree of variability in response to the stimulation, depending on the types and depths of anesthesia [24, 28, 31, 37, 40]. To examine the behavioral responses to FUS, without the confounding effects from anesthesia, experimentations in an awake setting are desired, and several recent studies on non-human primates and human subjects started to demonstrate the feasibility of tFUS in brain stimulation without the use of anesthesia [18, 20–23, 25, 41, 42].

We were motivated to develop a technique that will allow tFUS to be applied among unanesthetized, freely-moving small animals. Typically, a FUS transducer, much larger in size than the animal’s head, is maneuvered with optional image/visual-guidance for its stereotactic application during anesthesia [17, 24, 28, 30, 31, 37, 43]. To enable the experimentation in freely-moving small animals, one critical technical element is to make the transducer wearable. Accordingly, we developed a miniaturized, light-weight FUS transducer that can be worn (and detachable) by Sprague–Dawley rats (anesthetized) and demonstrated that FUS can be delivered to their primary somatosensory areas, with possibility for inducing long-term neuromodulatory effects [44]. A 3D-printed applicator that is designed to adjust the position of the transducer was attached to a pedestal, which was implanted onto the rat skull. The design enabled the individual adjustment of location/depth/orientation of the sonication focus. Recently, Li et al. [45] developed a dual-channel miniature FUS system that can stimulate two separate regions of the mice brain, and observed stimulation-mediated behaviors and extracellular neural action potentials. In their study, the transducers were surgically-fixed to the skull, which granted the use of the system among freely-moving mice. In the present study, we applied our wearable tFUS platform to stimulate motor cortical areas of freely-moving awake rats, and examined sonication-related behavioral responses from three different experimental conditions—(1) freely-moving awake status, (2) ketamine/xylazine anesthesia, and (3) isoflurane anesthesia. The response rates and latencies to the sonication were compared. After the completion of the sonication sessions, histological analysis was conducted on the rat brains to assess the presence of any undesirable tissue damage.

## Methods

### Ethical statement

All animal experiments were conducted under the approval of the local Institutional Animal Care and Use Committee.

### Preparation of the miniature FUS transducer/headgear

**Fig. 1:**
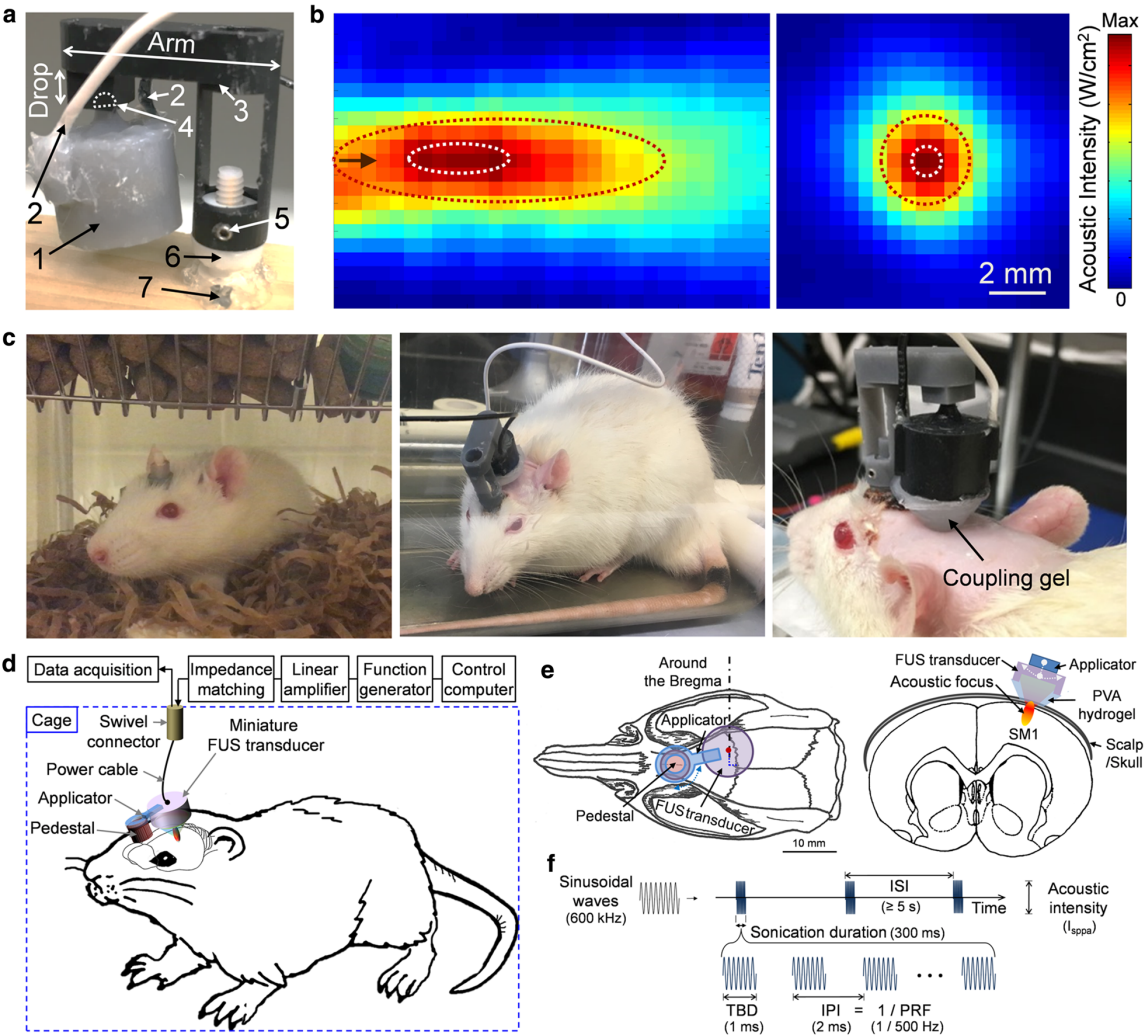
The schematics for the wearable miniature transcranial FUS headgear, acoustic profile, and experimental design. **a** A demonstration of the wearable setup applied on a wood-block. 1: FUS transducer, 2: power lines, 3: detachable applicator with customizable dimensions of ‘Arm’ and ‘Drop’, 4: ball-and-socket joint, 5: set screws to securely fix the applicator, 6: skull-mounted pedestal, 7: skull-mounted screws and medical glue. The drop length of the applicator in the photo was 4.5 mm. **b** The acoustic intensity profile across (left panel) the longitudinal plane and (right panel) the transversal plane at ~ 10 mm away from exit plane of the transducer. FWHM and FW90%M of the intensity profile are depicted with a red and white dotted line, respectively. The black arrow indicates sonication direction (from the left to right). Scale bar = 2 mm. **c** A rat resting in a cage (left panel), a freely-moving rat during the awake sonication session (middle panel), and an anesthetized rat (ketamine/xylazine) with a cone-shaped coupling hydrogel (right panel). **d** Schematic drawing of the experimental settings compatible with both anesthetized and freely-moving awake rat. **e** Exemplar targeting to the rat motor cortex for the left forelimb. **f** The sonication parameters used. *TBD* tone burst duration, *IPI* inter-pulse interval, *PRF* pulse repetition frequency, sonication duration, *ISI* inter-stimulation interval

A small (16 mm in diameter, 12 mm in height) and light (~ 6 g in weight) FUS transducer was built in-house (Fig. 1a) [44]. A disc-shape zirconate titanate (PbZr_x_Ti_(1−x)_O_3_; PZT) ceramic (American Piezo Ceramics, Mackeyville, PA) was used and fitted (air-backed) inside of a custom-built plastic housing. The plastic housing and back-lid of the transducer was designed (using CAD software; Solidworks Corp., Concord, MA) and printed by three-dimensional (3D) printing (Form2; FormLabs Inc., Somerville, MA). The back-lid of the transducer contained a ball-shape structure to fit the socket of an applicator (also 3D-printed), and held the transducer at a desired location/orientation (Fig. 1a). Both the transducer and applicator constituted the miniature tFUS headgear, and were attached to a pedestal (also 3D-printed), which was implanted on the skull of Sprague–Dawley rat (Charles River Laboratories, Wilmington, MA; see following section). Two set-screws were used to fasten the FUS headgear to the pedestal, ensuring a reproducible placement and orientation via lock-and-key mechanism. To accommodate the differences in individual neuroanatomy and cranial structures, applicators were customized with different ‘Arm’ and ‘Drop’ lengths (Fig. 1a).

### Surgical implantation of a pedestal on the rat skull

To apply the miniature tFUS headgear in a wearable form, a pedestal was surgically implanted on the anterior region of the rat’s skull. During the surgery, we measured the relative coordinates between the mounted pedestal and major skull anatomies (i.e., aural meatus, bregma, and lambda) to provide coordinates for the later FUS targeting. Two small screws were inserted (via burr holes) to the skull around the pedestal’s base to provide support along with a medical-grade adhesive (Loctite 18690; Henkel, Rocky Hill, CT). The skin around the pedestal (while exposing the top portion) was sutured back (using Vicryl 5-0 polyglactin 910 suture; Ethicon Inc., Somerville, NJ). After undergoing these surgical procedures, the rats were housed for at least 2 weeks to recover from the surgery prior to the tFUS sessions. The pedestal remained in place and provided long-term mechanical stability over 8 months.

### Actuation and characterization of the miniature FUS transducer

A fundamental frequency (FF) of 600 kHz was used to actuate the miniature transducer, and the acoustic intensity profile of the FUS transducer was characterized along the sonication direction as well as on the transversal plane at the focus (Fig. 1b). The detailed methods for the characterization process are described elsewhere [17]. The input signal was a sinusoidal wave generated by a function generator (33210A; Agilent, Santa Clara, CA) and amplified by a class-A linear amplifier (240 L; Electronics and Innovations Ltd., Rochester, NY) with an impedance-matching circuit. At the focus, the miniature transducer was capable of generating over 20 W/cm^2^ spatial-peak pulse-average intensity (I_sppa_). The acoustic focus was formed ~ 10 mm away from the exit plane of the transducer. The size of the focus, measured at full-width at half-maximum (FWHM) of acoustic intensity profile, was 11.5 mm in length and 3.0 mm in diameter. When it was measured at full-width at 90%-maximum (FW90%M), previously reported as the spatial dimension of the FUS-mediated neuromodulatory area [38, 39], the focal area was 3.5 mm in length and 1.0 mm in diameter.

### Acoustic coupling using PVA gel

A cone-shaped, polyvinyl alcohol (PVA) hydrogel (7–9% weight per volume; two freeze–thaw cycles, U228-08; Avantor, Center Valley, PA) was manufactured in-house for acoustic coupling between the transducer and scalp (Fig. 1c, right) (the detailed method can be found elsewhere [46]). The hydrogel showed negligible pressure attenuation on the order of 1%. A plastic cone [28, 32] or a bag [37, 39] containing degassed water has been typically used to couple the acoustic path, but could not be used for freely-moving awake animals due to the possibility of water escaping out of the coupling path/container depending on the rat’s dynamic behaviors (such as head-shaking and grooming).

### Animal preparation for tFUS sessions

For the tFUS sessions using anesthesia, the Sprague–Dawley rats (all male, *n *= 7) were anesthetized with either ketamine/xylazine (80:10 mg/kg; intraperitoneal; i.p.) or isoflurane (initial induction with 3–4% followed by 0.5% for the maintenance, at oxygen flow rate of 2 L per min; inhalation). An attempt was made to decrease the maintenance isoflurane concentration under 0.1%, as used by previous investigations in mice [28, 29], but rats emerged from the anesthesia prematurely, and therefore, not used in the present study. The fur on the head was shaved prior to each sonication to prevent any potential blocking of the sonication. The rats were then placed on a custom-built plastic platform in a prone posture with their limbs and tail freely hanging. After positioning the headgear and the accompanying PVA hydrogel, a generic ultrasound gel (Aquasonic; Parker Laboratories, Fairfield, NJ) was applied at each interface. Subsequently, we used the transducer geometry to estimate the virtual focal spot of sonication in space, and aligned the acoustic focus to the motor areas of the tail, limbs, or whiskers (Fig. 1e) while referencing the functional atlas of the rat motor cortex [47, 48]. Once an adequate level of anesthetic plane was detected, such as irregular breathing, the sonication session was conducted. We allowed for slight adjustment in the orientation of the transducer (Fig. 1a) for eliciting motor responses. Also, tFUS was intentionally delivered to off-target locations (lateral or caudal to the target, few millimeters away and including unilateral auditory areas) to examine the spatial specificity in stimulation. After each sonication session, the FUS headgear was removed, and the rats were returned to the housing facility for a minimum of 48 h before the next session (Fig. 1c, left).

To conduct the tFUS experiment in an awake state, we applied the same experimental procedures with the following steps. To shave the fur and apply the tFUS headgear (with the coupling hydrogel), the animals were lightly anesthetized using isoflurane (induction with 3–4%) for ~ 5 min. Then, the rats were moved to an empty cage and allowed to recover until they fully regained their pre-anesthetic behaviors (we determined that ~ 20 min was sufficient across the animals). No additional anesthesia was given to detach the FUS headgear from the pedestal.

### Experimental setup compatible with anesthetized/awake rats and data acquisition settings

**Fig. 2:**
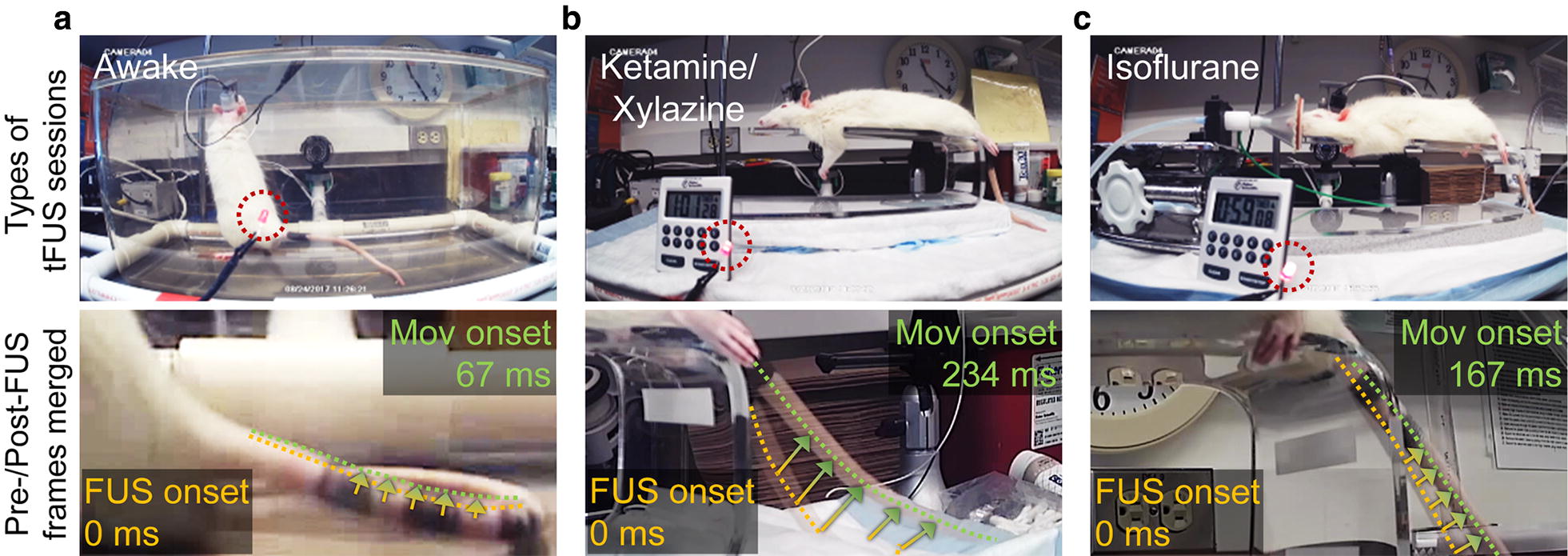
The experimental sessions (upper panels) and the merged images before/after tail movement (lower panels). **a** Freely-moving awake rats, as well as under light anesthesia of **b** ketamine/xylazine, or **c** isoflurane. The location of LED that shows the timing and duration of sonication is shown in dotted red circles. The movement onset (‘Mov onset’) latencies with respect to the FUS onset are also shown in the lower panels. The arrows indicate the elicited movement (see Additional files 1, 2, 3)

We established experimental setups that accommodated both anesthetized and awake rats. The schematics of the implemented wearable tFUS headgear, with the transducer actuation systems, are shown in Fig. 1d. A swivel connector (slip ring with flange-736; Adafruit, New York, NY) was located above the middle of the cage/platform, granting unrestricted motion and access to a power source for actuating the transducer during the awake tFUS sessions. A data acquisition system (PowerLab 8/30 and LabChart 7; ADInstruments, Colorado Springs, CO) was used to acquire time-series data of sonication events (onset timing and duration), being synchronized with a video recording (29.97 frames per second; FPS, by QTH44; Q-See; Anaheim, CA) to analyze the location and onset timing of the movement elicited by the sonication. Additionally, a light-emitting diode (LED), turned on in-sync with each sonication event, was placed within the field-of-view of the video recording as a visual indicator of the sonication timing (shown in Fig. 2a–c, upper panels).

### Sonication parameters for repeated tFUS sessions with anesthetized/awake rats

We conducted repeated tFUS sessions using a pulsed sonication scheme across all conditions. Based on our previous studies [37], we used the acoustic parameters (Fig. 1f) as follows: pulse repetition frequency (PRF) of 500 Hz, tone burst duration (TBD) of 1 ms (i.e., a duty cycle of 50%), and sonication duration of 300 ms, with a 5–10 s inter-stimulation interval (ISI), with varying acoustic output (see below). The sonication was administered to the motor areas in the left or right (side randomized) hemisphere of the rat brain. At the initial phase of this study, we gave stimulatory tFUS to each rat brain, starting from an acoustic intensity of 2.1 W/cm^2^ I_sppa_, increasing in increments of ~ 1 W/cm^2^, until the stimulatory response (i.e., movements from the tail, limbs or whiskers) was observed from the ketamine/xylazine as well as awake sessions. We determined that 14.9 W/cm^2^ I_sppa_ (for ketamine/xylazine anesthesia) and 8.8 W/cm^2^ I_sppa_ (for awake condition) were most suitable to elicit motor responses (regardless of their type) across all animals. These intensities were used in subsequent measurement of response rates. Acoustic intensity values at the target were estimated based on applying 17% of acoustic pressure attenuation through the rat skull [37].

### Response rates comparison across the repeated different anesthetic/awake conditions

We examined the response rates to the sonication from the same group of animals (*n* = 7, named as ‘R1’ to ‘R7’) through three repeated tFUS sessions, under each experimental condition. The sequence of these experimental sessions was randomized and balanced. Each tFUS session consisted of a total of 10 sonication events, targeting the tail, limb, or whisker motor areas in the brain. The individual animal’s mean response rates were compared using one-way analysis of variance (ANOVA) within each condition. The grand mean response rates were compared by repeated measures ANOVA and paired *t*-test across the conditions, with two-sample *F*-test for the equality of group variances.

### Analysis of the FUS-mediated movement location and onset latency

The location of FUS-mediated movement and the onset latency, across all the sonication parameters, were analyzed with high-resolution videos frame-by-frame using video analysis software (Quintic Player v29; Quintic Consultancy Ltd., Sutton Coldfield, UK) by three investigators. The onset of the tFUS was identified from the frame that showed the LED light turned on. A period greater than 500 ms before and after the tFUS onset (i.e., ≥ 15 frames) was examined for each sonication event. Only frames that showed distinctive movements were used to identify the type of movement and to measure the response latency with respect to the tFUS onset. Spontaneous movements from the body (for example, breathing-related movements) or a pattern of whisker movements were excluded to isolate stimulation-specific responses for the analysis.

### Examination of potential thermal effect

Potential thermal effect from the sonication was estimated using a formula of ΔT = 2αIt/(ρ_b_∙C_p_); where α = the absorption coefficient (0.014 cm^−1^ at ~ 600 kHz) [49], I = the intensity of ultrasound in the focal region, t = the ultrasound pulse duration, ρ_b_ = the density of brain tissue, and C_p_ = the specific heat of the brain tissue, where ρ_b_∙C_p_ is 3.796 J∙cm^−3^∙ °C [50, 51]. Using the equation, 0.016 °C was the estimated thermal increase, but considering a long ISI (≥ 5 s) (Fig. 1f) and subsequent heat dissipation, in conjunction with the small size of the acoustic focus, this temperature increase was considered to be negligible. An acoustic intensity level that corresponds to the mechanical index (MI) of 1.9, maximum allowed for diagnostic ultrasound device according to the food and drug administration (FDA)-guideline [52], was 46.5 W/cm^2^ I_sppa_ at 600 kHz.

### Post-sonication behavior monitoring and histological assessment

The biological effects of the repeated sonication sessions were examined across the experimental conditions (awake, ketamine/xylazine, and isoflurane). During the resting and survival periods after the sonication sessions, we regularly monitored the behavior and body condition of the animal for detecting any signs that indicated undesired neurological sequelae, including pain or distress. To examine the potential tissue damage, the animals were sacrificed at short-term (sacrificed within 0.7 ± 1.2 days; *n* = 3 rats) and long-term (41.5 ± 0.6 days; *n* = 4 rats) after the end of the last sonication session using the systemic cardiac perfusion of 10% formaldehyde (i.e., the method used to euthanize the animals) under ketamine/xylazine anesthesia, and the fixed brains were harvested. The brains were sectioned along the motor cortical areas, and the presence of hemorrhage, edema, ischemia, gliosis, inflammations were examined through histological analysis. Hematoxylin & eosin (H&E) staining was used to detect cell necrosis or local recruitment of inflammatory cells. Vanadium acid fuchsin (VAF)-toluidine blue staining was used to visualize ischemic neurons. Immunohistochemistry (IHC) of glial fibrillary acidic protein (GFAP) and caspase-3 staining were performed to examine glia infiltration or signs of neurodegeneration and to detect any apoptotic activity at and around the sonicated area, respectively. Two rats belonging to the short-term assessment underwent tail vein injection of the trypan blue dye, within 1 h after the end of the last sonication session to examine the presence of BBB disruption [13].

## Results

### Types of elicited responses from anesthetized/awake rats

The average weight of the same group of rats (*n* = 7, ‘R1–R7’) was 412.7 ± 33.8 g, 395.3 ± 55.0 g, and 388.3 ± 39.6 g (mean ± SD) in the awake, ketamine/xylazine, and isoflurane conditions, respectively (no significant differences, paired *t*-test, two-tailed, all *p* > 0.01). Table 1 shows the types of responses elicited by sonication from the wearable tFUS headgear across the conditions. The range of acoustic intensities used for the experiment was 2.3–14.9 W/cm^2^ I_sppa_ for the awake sessions, 7.5–14.9 W/cm^2^ I_sppa_ for the ketamine/xylazine sessions, and 9.0–14.9 W/cm^2^ I_sppa_ for the isoflurane sessions.

**Table 1:** FUS-mediated responses elicited during the awake (Aw), ketamine/xylazine (K/X), and isoflurane (Iso) conditions

Type of responses	Number of responsive rats		
	Aw	K/X	Iso
Tail/limbs/whiskers			
Whisker	7/7	7/7	4/7
Fore limb	5/7	7/7	6/7
Hind limb	3/7	6/7	6/7
Tail	7/7	4/7	5/7
Other responses			
Head/neck	7/7	7/7	–
Ears	7/7	1/7	–
Chewing	7/7	5/7	–

Across the experimental conditions, the number of responsive animals, out of 7 rats, was tabulated for each type of responses elicited by tFUS

The responses were observed above a certain threshold of acoustic intensities, i.e., 3.4 ± 1.8 W/cm^2^ I_sppa_ (mean ± SD, *n* = 7) for the awake condition, 10.2 ± 2.4 W/cm^2^ I_sppa_ (*n* = 7) for the ketamine/xylazine condition, and 12.4 ± 2.8 W/cm^2^ I_sppa_ (*n* = 6) for the isoflurane condition. The acoustic threshold levels from the awake condition were significantly lower than those observed from both anesthetic conditions (*t*-test, one-tailed, both *p* < 0.001) while there was no statistical difference between the ketamine/xylazine and isoflurane conditions (*t*-test, one-tailed, *p* > 0.05). Also, when tFUS was delivered to off-target locations (including auditory areas) or given under the effective I_sppa_, no responses were detected.

The elicited movements were seen from either of the tail/limbs/whiskers across all experimental conditons. These movements were similar with previous rodent studies involving ketamine/xylazine anesthesia [16, 31, 37]. We also observed twitches of the head/neck/ears and chewing behaviors in the awake and ketamine/xylazine conditions (listed as ‘other responses’ in Table 1), individually or accompanying the movements from the tail/limbs/whiskers. Under isoflurane anesthesia, the head/neck/ears movements and chewing behaviors were not seen. In terms of their qualitative evaluation, the range of the elicited movement was generally smaller in the case of the awake condition, than those observed from the anesthetic conditions (e.g., video-frame analysis from the tail response; Fig. 2a–c; Additional files 1, 2, 3). The head/neck/ears movements and chewing behaviors in the awake condition can be found in Additional files 4, 5 and 6.

### Response rates across the different conditions

**Fig. 3:**
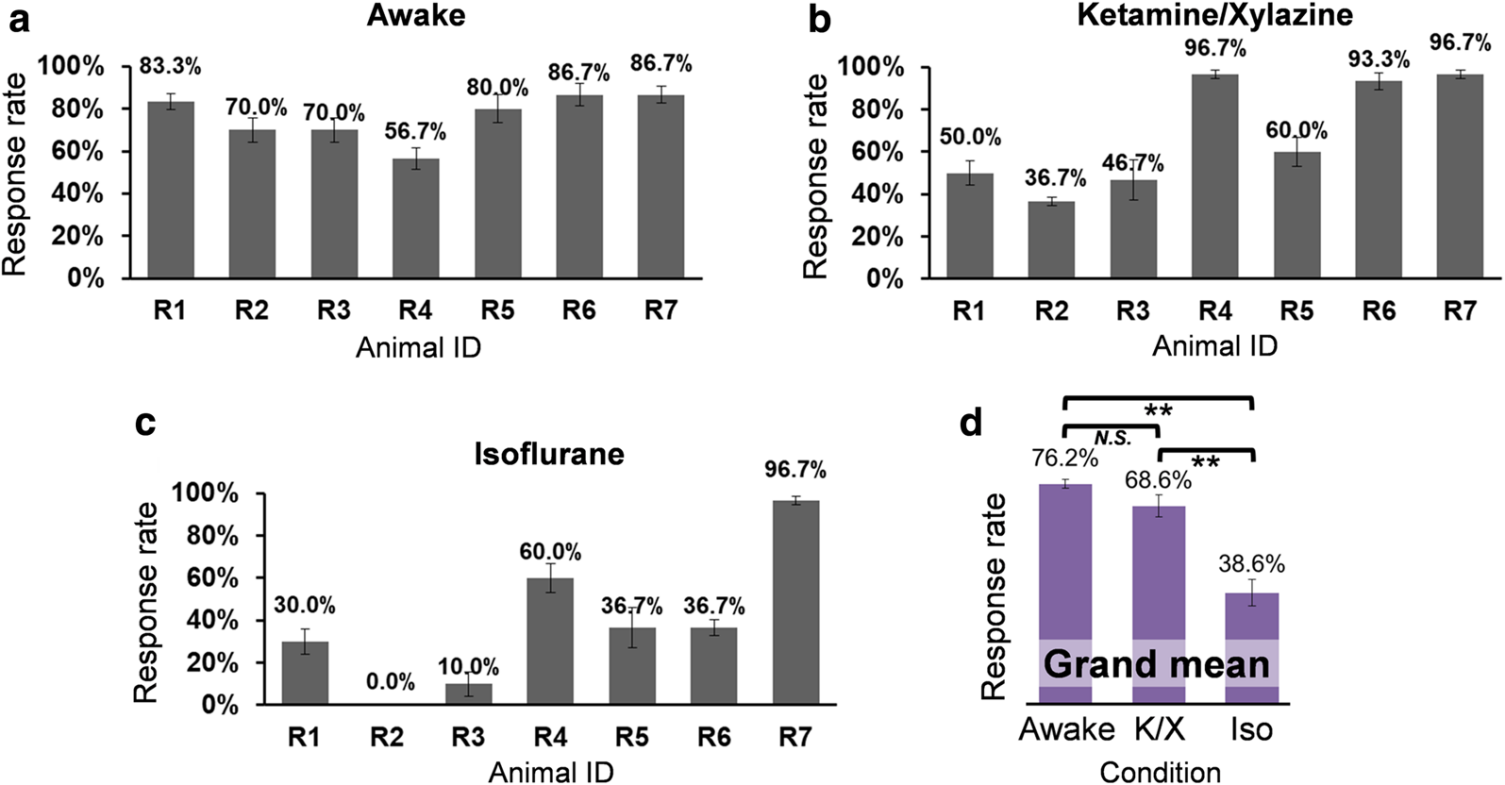
Response rates of the elicited movements by sonication from the wearable FUS headgear. **a**–**c** Each rat’s averaged response rate across three repeated sessions under each of the **a** awake, **b** ketamine/xylazine, and **c** isoflurane conditions. **d** Grand mean response rates across the same group of rats (*n* = 7 animals) under each experimental condition (paired *t*-test, one-tailed; **p ≤ 0.01, *N.S*., non-significant; *p* = 0.25). *K/X* ketamine/xylazine, *Iso* isoflurane

The response rate was calculated from each sonication session per each rat (‘R1’–’R7’), and averaged across three sessions. Each animal’s mean response rates (and its standard errors) are shown in Fig. 3 across the three different conditions of (1) awake (Fig. 3a), (2) ketamine/xylazine (Fig. 3b), and (3) isoflurane sessions (Fig. 3c). In the isoflurane condition, one animal (‘R2’) did not show any responses to the sonication.

The mean response rate in the awake sessions ranged 56.7%–86.7% while anesthetic conditions showed much wider ranges (i.e., 36.7%–96.7% in the ketamine/xylazine sessions and 0–96.7% in the isoflurane sessions). To evaluate the inter-animal variability in mean response rate, a one-way ANOVA was performed across the animals, and showed that the mean responses were not significantly different for the awake sessions (*p* = 0.25). On the other hand, during the anesthetic sessions, the ratio of FUS stimulation events resulted in motor response were significantly different among the animals (one-way ANOVA, *p* < 0.001 for both ketamine/xylazine and isoflurane conditions). Therefore, the data implicate that response rates were relatively even across the animals during the awake condition compared to those during the anesthetic conditions.

The overall response rate representing each condition was calculated by taking a grand mean of the response rates pooled from all rats (Fig. 3d), and revealed that both awake and ketamine/xylazine conditions showed significantly higher response rates than the isoflurane condition (repeated measures ANOVA*, p* < 0.05; augmented by paired *t*-test, one-tailed, *p* ≤ 0.01 for both awake and ketamine/xylazine sessions compared to the isoflurane sessions). Comparisons of the grand mean response rates between the awake and ketamine/xylazine sessions did not show statistical differences (paired *t*-test, one-tailed, *p* = 0.25). Meanwhile, the variability of the grand mean response rate (i.e., variances or dispersions) from the awake condition was significantly decreased compared to those from both anesthetic conditions (two-sample *F*-test, one-tailed, both *p* < 0.05), while there was no significant difference between the ketamine/xylazine and isoflurane sessions (*p* = 0.43).

### Onset latency of the elicited movements across the different conditions

**Fig. 4:**
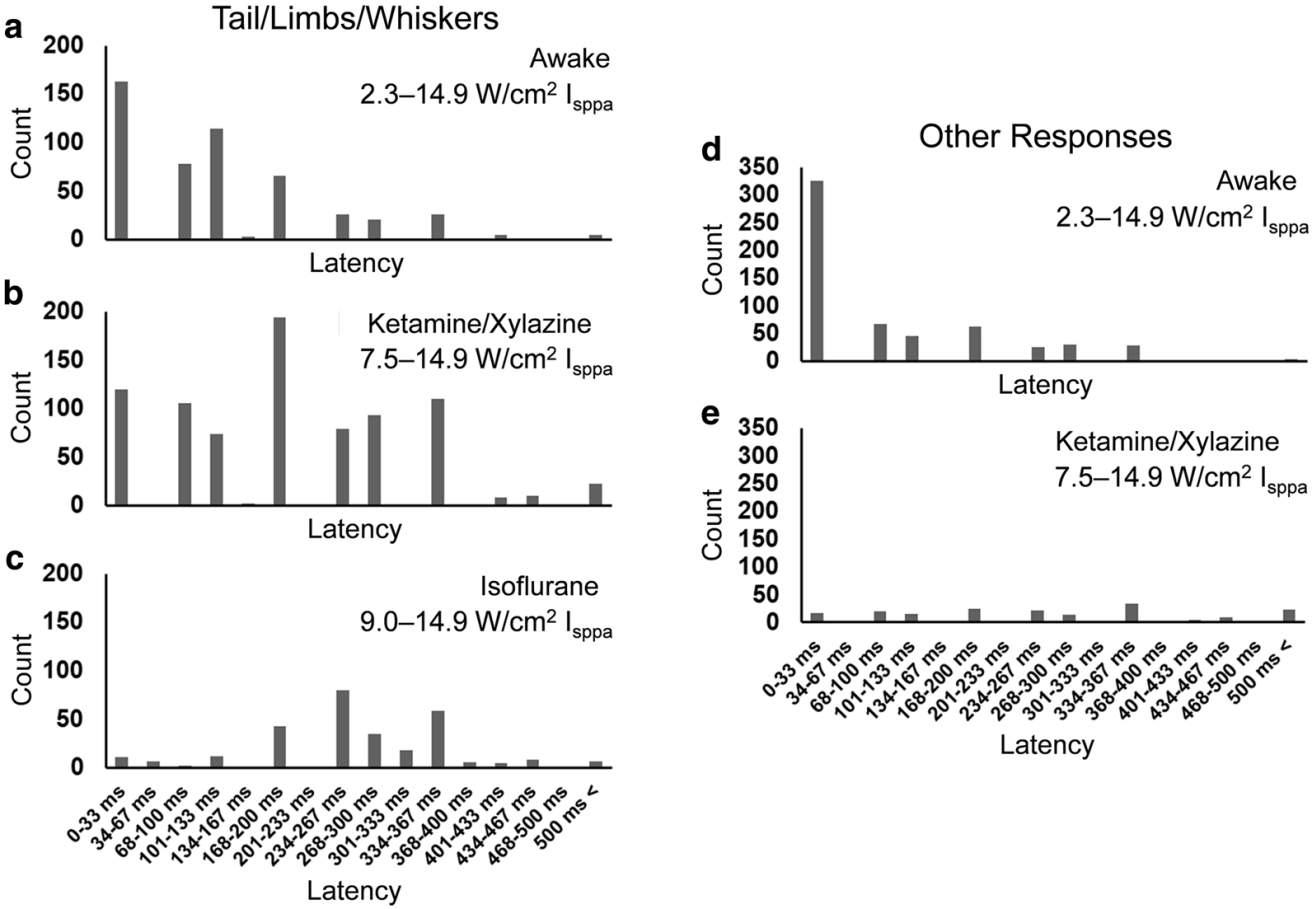
Onset latency histograms of the elicited responses after sonication onset timing (*n* = 7 rats). FUS-mediated tail/limbs/whiskers movement latencies in **a** the awake condition (from 510 sonication events), **b** ketamine/xylazine anesthesia (from 821 sonication events), and **c** isoflurane anesthesia (from 293 sonication events). The latencies of other responses from the head area including chewing behavior in **d** the awake condition (from 592 sonication events) and **e** ketamine/xylazine anesthesia (from 181 sonication events)

The number of events describing the successful tFUS stimulation (resulting in the movement of the tail/limbs/whiskers) and the onset latency were assessed for each condition using a histogram (Fig. 4a–c). Regardless of the experimental conditions, most (> 93%) of these responses were observed within a time frame of ~ 400 ms after the sonication onset. An average latency in motor responses was 139.1 ± 111.1 ms in the awake condition (*n* = 510), 212.8 ± 127.2 ms under ketamine/xylazine anesthesia (*n* = 821), and 282.9 ± 103.2 ms under isoflurane anesthesia (*n* = 293), while these latency values were significantly different to each other across the conditions (one-way ANOVA, *p* < 0.001; post hoc Tukey test, all *p* < 0.001). It is notable that the average latency of responses from the tail/limbs/whiskers in the awake condition was shorter than those under the anesthetic conditions.

In the awake and ketamine/xylazine conditions, we observed movements from the head/neck/ears as well as chewing behaviors (Table 1), and the same type of histogram showing its latency distributions was separately constructed (Fig. 4d and e; note that none were detected during the isoflurane sessions). The average latency of 111.9 ± 116.0 ms in the awake condition (*n* = 592) was also significantly shorter than the latency observed under ketamine/xylazine anesthesia (287.5 ± 178.0 ms; *n* = 181; *t*-test, one-tailed, *p* < 0.001).

To examine the presence of movement that is believed to be associated with acoustic startle responses (ASR) having short latencies (on the order of 10 ms [53–55]), we calculated the ratio of responses that occurred within 33 ms after the sonication onset (the limit of the video time frame based on 29.97 FPS), with respect to the total number of observed responses. For the tail/limbs/whiskers movements, the ratio was 32.0% in the awake condition, 14.6% under ketamine/xylazine anesthesia, and 3.8% under isoflurane anesthesia. For the head/neck/ears movements and chewing behaviors, the ratio was 55.1% in the awake condition, and 9.4% under ketamine/xylazine anesthesia. These data demonstrate that a greater portion of the responses occurred at a short latency range (< 33 ms) during the awake sessions.

### Post-sonication behavioral monitoring and histological analysis

**Fig. 5:**
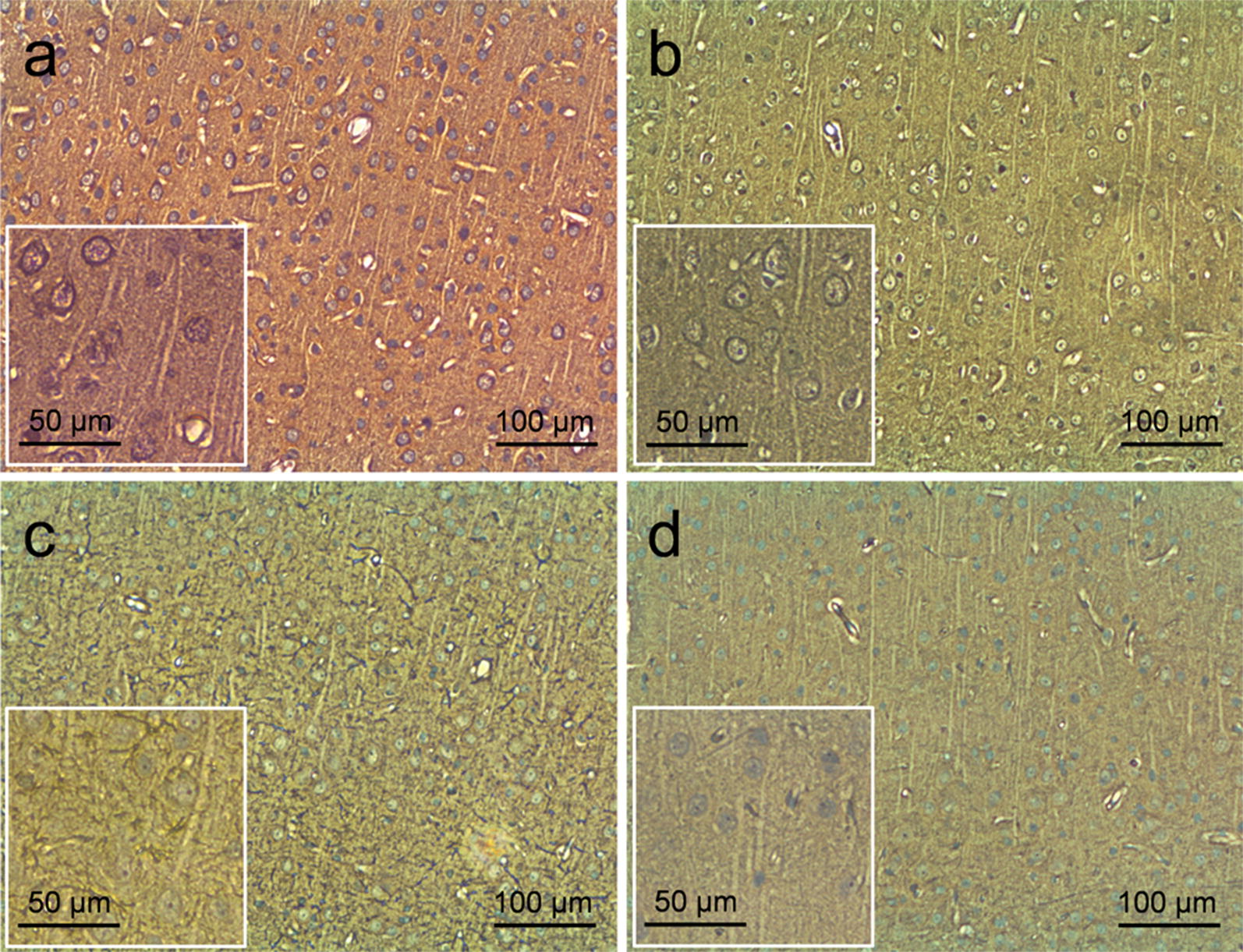
Exemplar histology results from the motor cortex of one rat. The staining (for ‘R6’) after the repeated sonication sessions with × 100 magnification (insets with × 200 magnification) of **a** H&E, **b** VAF-toluidine blue, **c** GFAP, and **d** caspase-3. The histology revealed that all the sonicated brain tissues were normal

All animals showed normal behavior and health status after the sonication experiments. The histological analysis (H&E, VAF-toluidine blue, GFAP, and caspase-3 staining) performed on the sonicated brain tissues at a short-term (0.7 ± 1.2 days, *n* = 3 rats) or long-term (41.5 ± 0.6 days, *n* = 4 rats) after the last FUS session showed no apparent signs of damage (Fig. 5 shows example slides from rat ‘R6’). The two rats that underwent the tail-vein trypan blue perfusion procedure did not show any signs of BBB disruption.

## Discussion

A miniature FUS transducer was developed in a wearable configuration and transcranially stimulated the motor cortical areas in rats. The transducer unit was attached to an implanted pedestal for each experimental session and detached prior to returning the rats to the animal housing. The location of the acoustic focus was adjusted by the transducer applicator, having different sizes (via 3D-printing) to fit the individual cranial anatomy of the rats. The setup enabled the tFUS experiments to be conducted repeatedly in both awake and anesthetized conditions (either i.p. injection of ketamine/xylazine or isoflurane inhalation). Subsequently, it allowed for systematic condition-specific comparisons of neuromodulatory outcomes, in terms of their physical representations, and response rates/variability with onset latencies. To our knowledge, this is the first study to demonstrate the efficacy of tFUS brain stimulation in awake rats, while having comparisons with two different anesthetic conditions.

### Types of elicited responses

The tFUS sonication elicited various physical motor responses across the study. Regardless of the experimental conditons, the elicited movements were seen from either of the tail/limbs/whiskers, demonstrating similiarities with previous rodent studies involving anesthesia [16, 28, 30, 31, 37]. In addition to these FUS-mediated movements, we also observed twitches from the head/neck/ears and chewing behaviors (which are new types of tFUS stimulation-related movement) in the awake and ketamine/xylazine conditions (listed as ‘other responses’ in Table 1). We conjecture that these new-found responses may be associated with the stimulation of corresponding motor areas due to the spatial proximity or overlap with intended motor regions for the whisker and forelimb [47, 48]. For example, imperfections in applying the sonication (e.g., mechanical slippage during application or due to the growth of cranium) can result in slight misalignments of the sonication target. Acoustic reverberation inside a small cavity of the rat skull [40, 56] with the potential to create multiple sonication foci may be another possible cause. It is also plausible that the twitches from the head/neck/ears and chewing behaviors were not seen in the previous studies due to the weight of transducer/coupling devices (water bags or plastic standoffs were used along with much bigger/heavier transducers), which became detectable in the present study using a light-weight wearable tFUS apparatus.

Under isoflurane anesthesia, a previous mice study [29] did report neck twitching behaviors, however, head/neck/ears movements and chewing behaviors were not seen in the present study. Although the definite causes for this discrepancy is difficult to ascertain, we conjecture that the given anesthetic setting (i.e., 0.5% isoflurane) did not allow sufficient motor neuron recruitment for overt movement. Provision of adequate anesthetic planes, e.g., accomodation of much lower isoflurane concentration using sophisticated anesthetic devices supported by body temperature control [28, 29], will allow for the further exploration of physical responses to tFUS stimulation.

### Acoustic intensity to elicit the responses

We found that thresholds existed, in terms of acoustic intensity, in eliciting motor responses. This is congruent with previous studies involving rodents [24, 28, 37] as well as in large animals [24] and in humans [21, 22]. The threshold acoustic intensity that started to elicit motor responses among the awake rats was much lower than those from anesthetic conditions. This finding is well-aligned with the notion that anesthesia generally suppresses neuronal excitability or dissociate the neural signal connectivity [57], which may elevate the threshold for excitation. The use of a lower acoustic intensity (in the awake condition), which will reduce overall dosimetry for the sonication, would be particularly advantageous for long and repeated FUS stimulation sessions.

### Qualitative examination of the range of the elicited movements

In terms of the qualitative evaluation of the range of the elicited movement, a tail movement, for example, was smaller in the case of the awake condition than those observed from the anesthetic conditions. We speculate that the observation may be attributed to the presence of residual muscle tension during awake state or the animal’s crawling postures that imposed weight to each of the limbs, which may hinder overt motor responses. Further study using measurements of strength of electromyography (EMG) or motor evoked potentials (MEP) is warranted to ascertain the electrophysiological information from FUS-mediated motor responses, especially in freely-moving awake animals.

### Response rates and their variability across the different conditions

We found that there were degrees of variability in the response rates among the animals and across the experimental conditions. Existence of such variabilities in the responsiveness were congruent with previous FUS-mediated studies reporting that the types/depths of anesthesia as well as individual differences can alter response rates [24, 28, 31, 37, 40]. Further analysis of inter-animal variability on response rates, measured from the movement data for the tail/limbs/whiskers, showed that the animals during the awake sessions manifested a more consistent level of responses compared to those during the anesthetic conditions. As to the causes for this reduced variability of responses in awake condition, individual-specific responsiveness/susceptibility to the anesthetic agents [57] as well as the method of its delivery (e.g., i.p. injection of ketamine/xylazine) might have played an important role. Regarding the grand mean response rate, although there were no statistical differences between the awake and ketamine/xylazine sessions, a significant difference did exist for the awake and isoflurane settings. Taken together, the awake condition offers the advantages of higher and more consistent/reproducible response rates compared to the anesthetic conditions.

### Onset latency of the elicited movements

Regarding the movement onset latency, most of the elicited responses, either from the tail/limbs/whiskers or from the head/neck/ears and chewing behaviors, were distributed within ~ 400 ms after the onset of the sonication event. An average latency in motor responses (from the tail/limbs/whiskers) was 139.1 ± 111.1 ms for the awake condition, 212.8 ± 127.2 ms for ketamine/xylazine, and 282.9 ± 103.2 ms for isoflurane. We note that the average onset latencies in awake rats were shorter compared to the ones from the anesthetic conditions, which may implicate that the use of anesthesia delays the onset timing of these elicited movements.

In the analysis of onset latency, intriguingly, a greater portion of responses were elicited within ~ 33 ms in the awake condition (over 30% for the tail/limbs/whiskers and over 50% for the head area) compared to below 15% in the anesthetic conditions. These responses having short latencies may be associated with the acoustic startle responses (ASR), known to be occurring within ~ 10 ms after the onset of the acoustic stimuli in rats [53–55]. Recently, Sato and colleagues reported a mice study that both ultrasound and audible sound showed similar brain activation patterns and motor response (consistent with a startle reflex) that were reduced by the chemical deafening of the animals [58], indicating that ultrasound may have an indirect link to acoustic-related (startle) effects and the elicitation of short latency responses. In this perspective, it is not surprising that awake animals, supposedly more susceptible to any external stimuli, showed a higher ratio of responses having short latencies than the anesthetic conditions. Wattiez and colleagues recently reported that cell-level acoustic neuromodulation occurs with an onset latency ≥ ~ 30 ms [42], lending further support to the idea that responses to the sonication below this latency could be related to startle effects. In the present study, most of the stimulation-related movements were observed at much longer latency, which cannot be explained solely by the ASR. In addition, the stimulation of the auditory areas did not produce any stimulation-related movement. Taken together, our data suggests that one should be aware of the presence of ASR-like phenomena, and exert caution when interpreting the physical responses to the acoustic stimulation.

### Technical limitations

In reviewing the execution of experimental settings, only the behavioral data was analyzed using video recording due to the lack of measurement of electrophysiological signals, such as EMG. As briefly discussed above, the small range of the elicited movements from awake animals made their detection difficult, which might have possibly contributed to the reduced response rates. These limitations warrant the integration of EMG measurement in future studies using freely-moving awake animals to ascertain the elicitation of the FUS-mediated motor responses. For enabling the EMG measurement from freely-moving awake animals, subdermal wires need to be implanted to the desired body/muscle parts (such as limbs or tail base) [59], whereby these wires are connected to a multi-channel electrode head pedestal that is compatible with our wearable tFUS headgear. Additional experimental modifications, such as the use of a high-speed camera, could also help to examine the response latencies with a higher time resolution.

We also note that the focal area, 3.5 mm in length and 1.0 mm in diameter measured at FW90%M of its intensity profile, can stimulate the brain regions outside the intended target (the motor cortex), reaching deeper brain structure. Since the present study did not have sufficient spatial resolutions in stimulating discrete rodent functional brain anatomy, the detailed effects of the stimulation on the response rate or the latencies could not be ascertained. We contemplate that use of large animal models (such as ovine, and corresponding larger neuroanatomy) will increase the relative spatial specificity of stimulation compared to that acquired from the rodent model, improving the assessment of region-specific effects of acoustic neuromodulation.

### Safety and non-thermal mechanism

In terms of the safety profile, all the animal behaviors were normal, with no brain damage or hemorrhage, after the repeated sonication sessions during a long-term period of ~ 5–8 months. In our previous rat study examining sonication parameters [37], H&E histology on a rat’s brain exposed to 22.4 W/cm^2^ I_sppa_ (corresponding to a spatial-peak temporal-average intensity of 11.2 W/cm^2^ I_spta_ with peak rarefactional pressure of 0.81 MPa, MI of 1.38) showed hemosiderin indicating potential earlier bleeding, while such signs were not observed in the present study with 14.9 W/cm^2^ I_sppa_ (7.5 W/cm^2^ I_spta_, 0.67 MPa, MI of 0.86). We conjectured that the use of longer ISIs (≥ 5 s vs. previously 2 s) and lower MI, with a miniature tFUS transducer having a smaller acoustic focus, compared to those used in the previous studies, possibly prevented the occurrence of sonication related brain hemorrhage. Also, the estimated potential thermal increase of 0.016 °C (see Methods), which is believed to be negligible considering heat dissipation during the ISI (≥ 5 s) and the small size of acoustic focus, supports that the biophysical mechanism behind the tFUS stimulation of neural cells could be linked with non-thermal mechanical factors [60]. The present work utilized the sonication parameters that are compliant with safety guidelines for the diagnostic ultrasound equipment (with an exception of the maximum MI of 0.23 for ophthalmological applications). However, we note that there is neither clear consensus nor the data on the sonication parameters (such as the acoustic intensity and the MI) for safe brain tissue stimulation. Further studies are, therefore, urgently needed to establish the safety guidelines for the acoustic neuromodulation.

## Conclusions

We demonstrated the application of FUS brain stimulation in a freely-moving rat model, utilizing a wearable tFUS headgear. The awake rats showed an increased response rate with reduced variability and shorter latency to FUS, in comparison with the neurostimulatory outcomes under the anesthetic conditions. Our analysis of latency distribution of responses suggests the possible involvement of ASR-like phenomena mixed with the stimulation-related physical movement. The use of small animal models, without confounding factors from anesthesia (including its unclear mechanism of action [57]), would be beneficial not only to gain further knowledge for reducing the variability (thus, may increase the reproducibility) in responsiveness to FUS but to gain more informative data regarding the potential presence of ASR. The ability to conduct FUS-mediated brain stimulation in awake small animals provides unprecedented opportunities for investigations that are not possible with anesthesia, such as sociobehavioral studies (e.g., self-administered brain stimulation [61]), or for the studies dealing with disease models that are influenced by anesthesia (e.g., epilepsy [33]).

## Additional files

**Additional file 1.** A movie showing rat tail movement in the awake experimental condition.

**Additional file 2.** A movie showing rat tail movement in the ketamine/xylazine anesthetic condition.

**Additional file 3.** A movie showing rat tail movement in the isoflurane anesthetic condition.

**Additional file 4.** A movie showing rat head/neck movement in the awake experimental condition.

**Additional file 5.** A movie showing rat ear movement in the awake experimental condition.

**Additional file 6.** A movie showing rat chewing behavior in the awake experimental condition.

## Abbreviations

DBS: deep brain stimulation
EpCS: epidural cortical stimulation
tDCS: transcranial direct current stimulation
TMS: transcranial magnetic stimulation
FUS: focused ultrasound
tFUS: transcranial focused ultrasound
BBB: blood–brain barrier
EEG: electroencephalographic
3D: three-dimensional
FF: fundamental frequency
FWHM: full-width at half-maximum
FW90%M: full-width at 90%-maximum
PVA: polyvinyl alcohol
FPS: frames per second
LED: light-emitting diode
PRF: pulse repetition frequency
IPI: inter-pulse interval
TBD: tone burst duration
ISI: inter-stimulation interval
ANOVA: analysis of variance
MI: mechanical index
FDA: food and drug administration
H&E: hematoxylin & eosin
VAF: vanadium acid fuchsin
IHC: immunohistochemistry
GFAP: glial fibrillary acidic protein
N.S.: non-significant
ASR: acoustic startle responses
EMG: electromyography
MEP: motor evoked potentials
Aw: awake
K/X: ketamine/xylazine
Iso: isoflurane
